# Leveraging radioisotope geochronology and diffusion chronometry to trace the thermal evolution of magmatic systems

**DOI:** 10.1093/nsr/nwaf141

**Published:** 2025-04-11

**Authors:** Wei-Ran Li, Fidel Costa

**Affiliations:** VRock Laboratory, Department of Earth Sciences, The University of Hong Kong, China; NWU-HKU Joint Centre of Earth and Planetary Sciences, Department of Earth Sciences, The University of Hong Kong, China; Institut de Physique du Globe de Paris, CNRS, Université Paris Cite, France

## Abstract

Time scales determined by radioisotope geochronology and diffusion chronometry for the same magmatic systems have shown variable degrees of discrepancy. We show in this paper that this could be due to the different temperature ranges that the two types of chronometers are effectively sampling, therefore combining them will help to trace the thermal evolution of magmatic systems on Earth and other planets.

Radioisotope geochronology provides fundamental constraints on the absolute ages of major events in Earth's geological and biological history. In comparison, diffusion chronometry determines durations of geological processes depending on the chemical heterogeneity within crystals or melts. The resolvable time scales of the two chronometers can differ greatly. For example, U-Pb and U-Th dating of zircon using secondary ion mass spectrometry (SIMS) or laser ablation-inductively coupled plasma mass spectrometry (LA-ICPMS) can produce ages with resolutions of 1–10 ka and precisions of 1%–2% relative, at spatial scales of less than tens of micrometres (see review by Schmitt *et al.* [1]). In comparison, the resolvable times of diffusion chronometers in magmatic systems are typically much shorter (from seconds to several thousand years) but can be prone to large uncertainties (see review by Costa *et al.* [2]). The time scales determined by the two approaches for the same magmatic systems have shown variable degrees of discrepancy (e.g. [3,4]). This could be partly due to the different temperature ranges in which these chronometers are effectively sampling, and is the main subject of this paper.

The temperature of magma can substantially vary over its lifetime and is a key variable that controls the diffusion coefficient (*D*) of elements in crystals and melts [5]. For a given element/isotope and mineral, the thermal evolution of the system can be assessed by considering three temperature (*T*) intervals and two key parameters (Fig. 1C):

- at high temperatures, diffusion is sufficiently fast that the entirety of the crystal and its environment are in equilibrium, i.e. the system is open, and time is not effectively recorded.
- with decreasing temperature and slower diffusion, the inner parts of the crystal may not be in equilibrium with its environment and thus it may start recording time and become chemically zoned in some elements/isotopes. The highest *T* at which this occurs can be expressed as the peak temperature (*T_peak_*) [6,7], whereas the lowest *T* is defined by the closure temperature at crystal rims [*T_c_*(rim)]. When *T_c_*(rim) < *T* < *T_peak_*, both chronometers record time, but the radioisotope chronometer can give a range of ages because the decay product can still diffuse into/out from the crystal.
- when *T* < *T_c_*(rim), the crystal becomes a closed system, and thus the diffusion chronometer stops recording whereas the radioisotope clock continues to be active.

**Figure 1.**
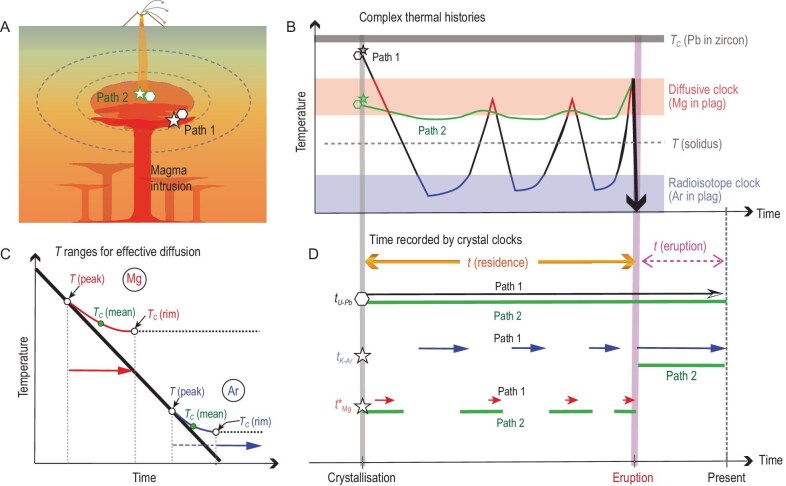
Schematic illustration of different time scales recorded by non-radioisotope/element (diffusion) and radioisotope (decay) chronometers due to different closure temperatures and thermal histories of the crystals. We consider two different thermal histories within a magma reservoir that is periodically intruded by less evolved magma from depth (panel A). The crystals (zircon shown as hexagons, and feldspar shown as stars) that reside in different parts of the reservoir (e.g. margins versus centre) are assumed to crystallize concurrently but have different *T-t* histories (panel B). The closure temperature of Pb in zircon and the *T* range where the Mg- and Ar-in-plagioclase clocks function, respectively, are shown as shaded areas in panel B (note that these temperatures vary with cooling rate and crystal size; see text and Supplementary Materials). The *T* ranges for effective diffusion (i.e. between *T_peak_* and *T_c_*(rim), marked in colorful solid lines) of Mg and Ar in plagioclase are illustrated in panel C (modified from [15]), assuming a constant cooling rate (marked in a thick solid line). The periods where the two chronometers are active are marked with arrows in different colours. Note that the K-Ar age could be non-zero before the temperature reaches *T_c_*(rim) (marked in blue dashed line; see text). Comparison of the time scales recorded by different crystal clocks along the two *T-t* paths is shown in panel D. The Mg diffusion time (t^*^_Mg_) and K-Ar age (*t_K-Ar_*) of plagioclase vary with the *T-t* paths, whereas the U-Pb age of zircon (*t_U-Pb_*) does not because of the very high closure temperature. We note that this figure shows simplified relationships and other factors, such as partial crystal dissolution, partial resetting of the K-Ar ages and grain sizes, could change the details but not the general systematics.

The equation commonly used to understand these relationships defines the mean *T_c_* for the whole crystal ($\overline {{T}_c} $; [8]):


(1) \begin{equation*}
\frac{E}{{R\overline {{T}_c} }} = \ln \left[ { - \frac{{AR{{\overline {{T}_c} }}^2{D}_0}}{{E\ \left( {dT/dt} \right)\ {a}^2}}} \right],
\end{equation*}

where ${D}_0$ and $E$ are the pre-exponential factor and activation energy of *D* in the Arrhenius equation [e.g. $D = {D}_0{\mathrm{exp}}( {\frac{{ - E}}{{RT}}} )$], $A$ is a geometric factor, $a$ is the characteristic diffusion distance, $dT/dt$ is the cooling rate, and *R* is the gas constant. Notably, Equation (1) uses a mean $\overline {{T}_c} $ which differs from a hypothetical *T_c_* that would decreases from the crystal core to rim. The relationship between these different temperatures is such that *T_peak_* ≥ *T_c_*(core) > $\overline {{T}_c} $ > *T_c_*(rim) (Fig. 1C), and they can have quite different values. Here we use the Mg-in-plagioclase diffusion chronometer as an example. Applying the parameters that Faak *et al.* [7] used to estimate *T_c_*(rim) ≈ 660°C, we calculate *T_peak_* ≈ 1100°C (using Equation (6) of [7]) and $\overline {{T}_c} $ ≈ 1000°C (using Equation (1) above; see details in Supplementary Materials). This diffusion chronometer is thus active from *T_peak_* which could be close to the liquidus of a mafic magma to *T_c_*(rim) which may be sub-solidus. Measuring (sub-)micrometre scale concentration gradients at crystal rims using high-precision-spatial-resolution analytical techniques (e.g. NanoSIMS) may give even lower values of *T_c_*(rim).

The idea of combining different radioisotopes-minerals with different closure temperatures is the basis of thermochronology as has been widely applied to study low-to-moderate temperature near-surface/upper crustal processes. However, it is more challenging to apply to moderate-to-high temperature magmas, due to the more restricted range of *T* recorded by radioisotopes in minerals and the complex temperature-time (*T-t*) paths of fluid-dynamical magmatic systems. Taking zircon and feldspars as examples, the $\overline {{T}_c} $ of Pb and Th in zircon are calculated to be ∼1070–1280°C and ∼1430–1660°C, respectively, whereas those of Ar in plagioclase and K-feldspar are much lower (∼490–890°C and ∼280–400°C, respectively; see detail in Supplementary Materials). The difference in the $\overline {{T}_c} $ of feldspars and zircon could lead to ^40^Ar/^39^Ar feldspar ages that are tens to hundreds of thousands of years younger than U-Pb (or Th) ages of zircons, as reported in many studies (e.g. [9,10]). The calculated $\overline {{T}_c} $ of Pb and Th are much higher than the saturation temperature of zircon (e.g. usually <800°C in granite); at such high temperatures a pre-existing crystal would completely dissolve within short durations (e.g. of ∼hours to ∼days; [11]). Therefore, these closure temperatures are fictive and have a minimal effect on the zircon dates.

The age ranges determined by multiple radioisotopes-minerals have been combined with an improved understanding of diffusion kinetics to unravel the thermal conditions of magma storage and eruption. Recent studies of large and silica-rich eruptions have found that the ^40^Ar/^39^Ar dates of plagioclase/sanidine can exceed those of the U-Th disequilibrium (e.g. [12]) and U-Pb dates of zircon (e.g. [4]). This was interpreted to be due

to varying degrees of retention of radiogenic ^40^Ar in a magmatic system that was in a cold storage regime, followed by incomplete degassing at/after the eruption. This was also supported by an exponential distribution of the feldspar ages that followed a survival function [4,12]. Diffusion modelling of Ar in sanidine antecrysts indicates long-term cold storage at sub-solidus conditions [4], which are cooler than the near-solidus condition previously suggested for silica-rich crystal mushes (e.g. [3]). These findings create new challenges to understanding and modelling the processes required for rejuvenating fully solidified magma bodies towards eruption and reframe the volcanic-plutonic connection.

The radioisotope-only chronometry can be further combined with single/multiple non-radioisotope/element diffusion chronometry, to gain new and broader insights into the thermal history of magmatic systems. We illustrate this in Fig. 1, where we consider two possible *T-t* paths with different heating-cooling histories within a large magma body. One path is for the system's margins which may be close to the intrusion sites and experience large thermal fluctuations before eruption (path 1 in Fig. 1), whereas the other is for the interior of the system that may be more thermally buffered and experience limited thermal variations over time (path 2 in Fig. 1). We illustrate the differences in the ^40^Ar/^39^Ar ages and Mg diffusion times of plagioclase and the U-Pb ages of zircon along the two paths. Plagioclase crystals stored at low temperatures for long durations may record short diffusion chronometry times but old ^40^Ar/^39^Ar apparent ages; the two records complement each other but do not record the entire thermal history due to differences in *T_c_* (Fig. 1B). In contrast, plagioclase crystals stored at hotter conditions for a longer time may record longer diffusion chronometry times but may provide younger ^40^Ar/^39^Ar ages similar to that of eruption. Therefore, we suggest that a sensible estimation of magma storage time may be obtained from a large number of crystals that may collectively record most, if not all, of the thermal history of the magma. Specifically, the relatively hot periods may be revealed by plagioclase crystals showing diffusion-induced zoning (e.g. Mg, Sr, Ba) and young ^40^Ar/^39^Ar ages close to the eruption age; the latter may be estimated from Bayesian analyses of the ^40^Ar/^39^Ar dates (e.g. [13]), and/or independently determined by other radioisotope systems of lower *T_c_* (e.g. U-Th/He in zircon; [14]). The relatively cold periods may be recorded by plagioclase crystals showing older ^40^Ar/^39^Ar dates and less pronounced diffusion-induced chemical zoning. Zircon crystals with different thermal histories are likely to provide equivalent/similar ranges of U-Pb (or Th) ages, although those that have experienced large temperature fluctuations may show crystallization gaps or missing records depending on the saturation temperature. Given the large variety of elements-isotopes that can be accessed with diffusion chronometry, as well as the increasing precision of radioisotope dating, it should be possible to recover the full *T-t* paths of magma evolution if sufficient crystals from a given magma body are investigated.

In summary, we show that radioisotope and diffusion-based chronometers can provide complementary records of the *T-t* paths of magma. The records in different crystals from the same eruption deposits are subject to the pre-eruptive thermal histories which can greatly differ within a large magma body, thus sampling and analyzing a large number of crystals is necessary. Leveraging combined chronometry and high-precision-spatial-resolution analytical techniques will provide new insights into not only the timing of geological events in the Earth's history, but also the thermal evolution of magma bodies on Earth, Moon and other rocky planets.

## Supplementary Material

nwaf141_Supplemental_File

## Data Availability

The calculated closure temperatures and peak temperatures, as well as parameters used in the calculation, are available in the online Supplementary Material. The python code we developed for the calculation is archived in a Zenodo repository (https://doi.org/10.5281/zenodo.15015271) and updates will be provided at: https://vrocklab.hku.hk/python-code.
